# Matrix Factorization-Based Prediction of Novel Drug Indications by Integrating Genomic Space

**DOI:** 10.1155/2015/275045

**Published:** 2015-05-19

**Authors:** Wen Dai, Xi Liu, Yibo Gao, Lin Chen, Jianglong Song, Di Chen, Kuo Gao, Yongshi Jiang, Yiping Yang, Jianxin Chen, Peng Lu

**Affiliations:** ^1^Institute of Automation, Chinese Academy of Sciences, Beijing 100190, China; ^2^Beijing University of Chinese Medicine, Beijing 100029, China

## Abstract

There has been rising interest in the discovery of novel drug indications because of high costs in introducing new drugs. Many computational techniques have been proposed to detect potential drug-disease associations based on the creation of explicit profiles of drugs and diseases, while seldom research takes advantage of the immense accumulation of interaction data. In this work, we propose a matrix factorization model based on known drug-disease associations to predict novel drug indications. In addition, genomic space is also integrated into our framework. The introduction of genomic space, which includes drug-gene interactions, disease-gene interactions, and gene-gene interactions, is aimed at providing molecular biological information for prediction of drug-disease associations. The rationality lies in our belief that association between drug and disease has its evidence in the interactome network of genes. Experiments show that the integration of genomic space is indeed effective. Drugs, diseases, and genes are described with feature vectors of the same dimension, which are retrieved from the interaction data. Then a matrix factorization model is set up to quantify the association between drugs and diseases. Finally, we use the matrix factorization model to predict novel indications for drugs.

## 1. Introduction

The number of new drugs introduced to the market has significantly declined in the past decade as the procedure of drug approval is time-consuming, costly, and risky. It is estimated that an average of more than $800 million is spent in a time period of 15 years to bring a single drug to market [1]. Therefore, there has been rising interest in alternative drug indication discovery, which is an effective strategy of drug development. It can renew a failed drug or expand the number of indications for a successful one. Besides, this strategy offers the possibility of reducing research and development timelines without increasing risk [2]. The successful stories of a novel indication of a drug for a new condition include Minoxidil, Viagra, Avastin, and Rituxan [3]. However, most of these discoveries are based on serendipity and not systematic analysis.

Recently, many systematic computational techniques have been proposed to generate new hypotheses for additional indications of drugs. Most of the attempts have focused on either drug repositioning or matching drug and disease profiles. Gottlieb et al. retrieved 5 drug-drug similarity measures and 6 disease-disease similarity measures to learn a logistic regression classifier, which performs well in predicting potential drug-disease associations [4]. Iorio et al. utilized transcriptomics to discover new drug mode of action [5]. Li and Lu took advantage of pathway information [6] to infer new uses of existing drugs. Yang and Agarwal selected clinical side effects to undertake systematic drug repositioning [7]. Researchers utilize the rich information from these profiles for the large scale prediction of novel drug indications.

Clearly, the previous research could help reposition a known drug to a new indication. However, most of these methods paid more attention to the creation of explicit profiles of drugs and diseases. For example, Gottlieb et al. prepared five features for drugs and two sets of features for diseases [4] in order to calculate similarities for drugs and diseases, respectively. In fact, it takes time to collect these profiles before establishing the model. Furthermore, the selection of features is not an easy job, which needs lots of domain knowledge and experience.

To get rid of the concerns in preparing substantial profiles for drugs and diseases, we present an alternative means by taking advantage of the interaction data. As we know, matrix factorization is the frequently used technique in collaborative filtering, which predicts user preferences for product by learning past user-item relationships [8]. In fact, previous studies have also adopted interaction data in pharmaceutical sciences. For example, Zheng et al. used matrix factorization to predict effective drug-target interaction from the known ones [9]. Gönen proposed a Bayesian matrix factorization model for predicting drug-target interaction networks [10]. Nevertheless, seldom research focuses on adopting known drug-disease associations in generating matrix factorization models for the prediction of novel drug indications. In this work, we propose a matrix factorization model with the integration of genomic space to detect potential drug-disease associations and predict novel drug indications. Specifically, genomic space here refers to interactions regarding genes, including drug-gene interactions, disease-gene interactions, and gene-gene interactions. The introduction of genomic space can add molecular biological information for predicting drug-disease associations [11]. In fact, genomic topology has also played a role in previous research. Zhao and Li [12] utilized important gene modules to decipher how drugs and diseases are associated in the molecular level. Li et al. [13] employed network interactions in the molecular level to evaluate synergies between drugs. In our work, we use eigenvalue decomposition to calculate feature vectors of genes from gene interaction network. In this way, the topological characteristics of gene interaction network are retrieved for investigation of drug-disease associations.

**Figure 1:**
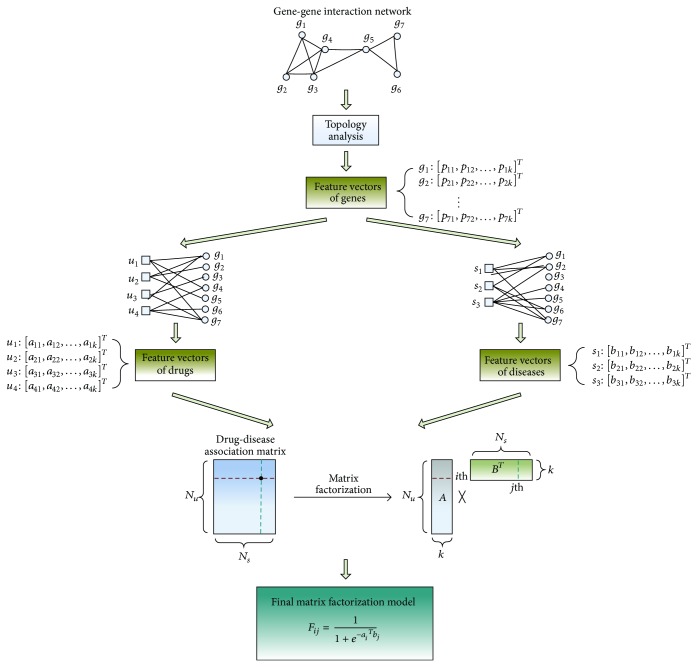
Strategy pipeline. Firstly, feature vectors of genes are extracted from gene interaction network. Then, feature vectors of the same rank are obtained for drugs and diseases from drug-gene interactions and disease-gene interactions, respectively. Next, a matrix factorization model is generated to reconstruct the known drug-disease associations. Finally, the estimated feature vectors of drugs and diseases are used to infer new drug-disease associations and predict novel drug indications.

The workflow of our strategy is illustrated in Figure 1; low-rank feature vectors of genes are firstly retrieved from gene interaction network by using eigenvalue decomposition. Then feature vectors of drugs and diseases are, respectively, obtained from drug-gene interactions and disease-gene interactions. In this way, the topological characteristics of gene interaction network are fused into the features of drugs and diseases. Afterwards, a matrix factorization model is generated to approximate the known associations between drugs and diseases. The output of the model can be regarded as a measurement of the possibility of association between one certain drug and one certain disease. Finally, the matrix factorization model is used to infer new drug-disease associations and predict novel drug indications. In this paper, we firstly demonstrated the role of genomic space in our strategy by comparing matrix factorization models with genomic space and without genomic space. Then our strategy was compared with three state-of-the-art collaborative filtering tools to demonstrate a better performance of our strategy. With the accumulation of more and more interaction data in the future, we believe that our method would play a greater role.

## 2. Methods

### 2.1. Problem Definition

Let *U* = {*u*_1_, *u*_2_,…, *u*_*N*_*u*__} be a given set of drugs, *S* = {*s*_1_, *s*_2_,…, *s*_*N*_*s*__} be a given set of diseases, and *G* = {*g*_1_, *g*_2_,…, *g*_*N*_*g*__} be a given set of genes, where *N*_*u*_, *N*_*s*_, and *N*_*g*_ are the number of drugs, the number of diseases, and the number of genes, respectively. We use *Y* to denote the interaction data. Specifically, let *Y* ^*US*^ be a *N*_*u*_ × *N*_*s*_ binary matrix of true labels of drug-disease associations, in which only some entries are known. In the known entries, *Y*_*ij*_^*US*^ = 1 if drug *u*_*i*_ and disease *s*_*j*_ are known to be associated with each other and *Y*_*ij*_^*US*^ = 0 if drug *u*_*i*_ and disease *s*_*j*_ are known to be not associated. The other entries are unknown as we are not sure whether they are associated or not. Then we use two binary matrices to represent the associated genes of drugs and diseases: let *Y* ^*UG*^ be a *N*_*u*_ × *N*_*g*_ binary matrix of drug-gene interactions, where *Y*_*ij*_^*UG*^ = 1 if drug *u*_*i*_ and gene *g*_*j*_ interact with each other, and *Y*_*ij*_^*UG*^ = 0 otherwise; let *Y* ^*SG*^ be a *N*_*s*_ × *N*_*g*_ binary matrix of disease-gene interactions, where *Y*_*ij*_^*SG*^ = 1 if disease *s*_*i*_ and gene *g*_*j*_ interact with each other, and *Y*_*ij*_^*SG*^ = 0 otherwise. Finally, the gene interaction network, consisting of *N*_*g*_ genes, is adopted for exploiting genomic topology. To study the effect of the integration of genomic space, we assume that all the *N*_*u*_ drugs and all the *N*_*s*_ diseases have interacting genes in the gene interaction network.

The goal is to calculate a *N*_*u*_ × *N*_*s*_ scoring matrix *F*, which is derived from the known interaction data, to predict scores for the unknown ones in *Y* ^*US*^. Specifically, the entry *F*_*ij*_ indicates the potential of association between drug *u*_*i*_ and disease *s*_*j*_.

### 2.2. Creation of Feature Vectors

The introduction of genomic space is aimed at providing biological information in the molecular level for predicting drug-disease associations. The main idea is to exploit the genomic space to create low-rank feature vectors, which are used to describe the feature spaces of genes, drugs, and diseases. Firstly, we construct a gene interaction network from the gene-gene interactions. The network is composed of a certain number of gene nodes and a set of edges describing the interactions between gene nodes. To extract topology information from the network, the gene closeness metric is proposed. Specifically, for each pair of gene nodes (*g*_*i*_, *g*_*j*_) where *g*_*i*_ ∈ *G* and *g*_*j*_ ∈ *G*, the gene closeness metric is computed by

(1) $$\begin{array}{c} c_{ij}=a^{\prime }\exp^{-b^{\prime }d_{ij}}, \end{array}$$

where *d*_*ij*_ is the shortest distance between *g*_*i*_ and *g*_*j*_ in the network and *a*′ and *b*′ are the adjustment parameters. It should be noted that *d*_*ij*_ is regarded as infinity when the two gene nodes are unreachable. The gene closeness is used to measure the relationship of two genes that belong to the set *G*. In this way, a *N*_*g*_ × *N*_*g*_ gene relation matrix *C* is generated from the genomic space. Afterwards, eigenvalue decomposition [14] of the matrix *C* is applied to construct a unified space, which is a low-rank Euclidian space, so that all the *N*_*g*_ genes can be represented by sets of *k*-dimension feature vectors {**p**_*g*_*i*__}_*i*=1_^*N*_*g*_^. The detailed procedure is as follows.

1. Calculate the eigenvalues, which are arranged in the descending order, and the corresponding eigenvectors of the gene relation matrix *C*.
2. Retrieve the first *k* eigenvalues to form the *k* × *k* diagonal matrix Λ; retrieve the corresponding *k* eigenvectors to form the *N*_*g*_ × *k* matrix Γ, where each column is an eigenvector.
3. Calculate the *N*_*g*_ × *k* matrix *P* = ΓΛ^1/2^, by which the gene relation matrix *C* can be decomposed as *C* ≈ *PP* ^*T*^ = ΓΛ^1/2^Λ^1/2^Γ^*T*^.
4. Represent all the genes by using the row vectors of the matrix *P* = (**p**_*g*_1__, **p**_*g*_2__,…, **p**_*g*_*N*_*g*___)^*T*^.

In this way, the characteristic of gene interaction network is extracted to form the feature vectors **p**_*g*_ which constitute the feature space of genes.

Next, the feature spaces of drugs and diseases are, respectively, obtained according to the interaction matrices *Y* ^*UG*^ and *Y* ^*SG*^. We calculate the feature vectors of drugs and diseases, denoted as **p**_*u*_ and **p**_*s*_, respectively, using the following formulae:

(2) pui∑j=1NgYijUGpgj∑j=1NgYijUGi=1,2,…,Nupsi=∑j=1NgYijSGpgj∑j=1NgYijSGi=1,2,…,Ns.

After that, feature vectors of genes, drugs, and diseases are integrated into a unified space of the same rank. The assumption under this integration is that the association between drug and disease has its evidence in the gene interaction network. In other words, if a drug is known to be associated with a disease, then targets of the drug might be more highly interconnected with the disease genes in the interactome network.

### 2.3. Generation of Matrix Factorization Model

The intuition behind the proposed matrix factorization model is that there should be some latent features that determine how drugs and diseases are associated. As illustrated in Figure 1, the *N*_*u*_ × *N*_*s*_ matrix *Y* ^*US*^ is factorized into two low-rank matrices *A* and *B*: a *N*_*u*_ × *k* feature matrix of drugs denoted as *A* = (**a**_1_, **a**_2_,…, **a**_*N*_*u*__)^*T*^ and a *N*_*s*_ × *k* feature matrix of diseases denoted as *B* = (**b**_1_, **b**_2_,…, **b**_*N*_*s*__)^*T*^. In matrix *A*, the *i*th row **a**_*i*_^*T*^ represents the feature vector of the *i*th drug; in matrix *B*, the *j*th row **b**_*j*_^*T*^ represents the feature vector of the *j*th disease. To integrate the genomic space, the original states of matrices *A* and *B* are obtained from feature vectors of drugs and diseases, which are extracted from the gene interaction network:

(3) Aa1,a2,…,aNuT=pu1,pu2,…,puNuTBb1,b2,…,bNsT=ps1,ps2,…,psNsT.

By using *Y*_*ij*_^*US*^ = **a**_*i*_^*T*^ **b**_*j*_, we can reconstruct the known associations between drugs and diseases and predict possibilities for the unknown ones. In order to constrain the output to fall within the range of 0-1, we add a sigmoid active function to the output *Y*_*ij*_^*US*^. Thus, the final model is described as

(4) $$\begin{array}{c} F_{ij}=f\left(\;{a_{i}}^{T}b_{j}\;\right)=\frac{1}{1+\exp^{-{a_{i}}^{T}b_{j}}}, \end{array}$$

where *F*_*ij*_ is the predicted possibility of the association between the *i*th drug and *j*th disease, making up the scoring matrix *F*.

To estimate *A* and *B* in the matrix factorization, we choose to minimize the squared error of the known drug-disease associations. To avoid overfitting of *A* and *B* to the known associations, an *L*2 regularization is added to the loss function, which can be written as follows:

(5) LA,B=argminA,B⁡12·∑i,j∈RYijUS−faiTbj2+λai22+bj22,

where *ℜ* is the set of known drug-disease associations and *λ* is a regularization coefficient. In the optimization function, *Y*_*ij*_^*US*^ is the true label for association between the *i*th drug and the *j*th disease, while *f*(*a*_*i*_^*T*^ *b*_*j*_) is the predicted value for the corresponding drug-disease pair.

To learn the model, we use stochastic gradient descent to estimate *A* and *B*, which minimizes the optimization function. By setting the partial derivative of *L* as ∂*L*/∂**a**_*i*_ = 0 and ∂*L*/∂**b**_*j*_ = 0, we obtain the updating rule of *A* and *B* as follows:

(6) aiai+ηei,jbjFij1−Fij−λaibj=bj+ηei,jaiFij1−Fij−λbj,

where *e*_*i*,*j*_ = *Y*_*ij*_^*US*^ − *F*_*ij*_ is the difference between the true value and the predicted value and *η* is the learning rate.  Algorithm 1 uses pseudocode to show the optimization process of the matrix factorization model with stochastic gradient descent. We stop the training until the model converges to a relatively stable state.

## 3. Results

### 3.1. Preparation of Interaction Data

The interaction data, including drug-disease associations, drug-gene interactions, and disease-gene interactions, were firstly manually collected from [4, 15, 16]. Then two genes are considered to be interacted if their products interact with each other in the protein-protein interaction network, which is retrieved from HPRD database [17]. In this way, gene-gene interactions were acquired to yield a gene interaction network of 36882 unique pairwise binary interactions between 9415 genes. We unified these data sources by using DrugBank ID to represent drugs, using OMIM ID to represent diseases, and using Entrez Gene ID to represent genes. To meet the requirement that all drugs and diseases in the drug-disease associations should have interacting genes in the gene interaction network, we examined the drug-disease associations and kept the ones in which both the drug and the disease have interactors in the 9415 genes. In this way, 213 drug-disease associations involving 130 drugs and 50 diseases were kept. Besides, 776 drug-gene interactions concerning the 130 drugs and 74 disease-gene interactions concerning the 50 diseases were left. In total, there were 994 distinct genes in these interactions. The following research was carried out based on these data.

### 3.2. Establishment of Our Model

To extract topology information from gene interaction network, gene closeness metrics were computed for the 994 genes based on the interactome network of 9415 genes to form a 994 × 994 gene relation matrix. During the computation, we set *a*′ = 10 and *b*′ = 0.25 to make the values of gene closeness relatively evenly distributed. It should be pointed out that the selection of *a*′ and *b*′ is not a key issue in the research, as it is a simple transformation of the values and has a limited influence on our method. The matrix was used to extract feature vectors of the 994 genes. Then features of drugs and diseases were retrieved according to the drug-gene interactions and disease-gene interactions. Next, training set of drug-disease pairs was prepared beforehand. The positive set was made up of the 213 known drug-disease associations. The negative set was generated by randomly combining drugs from the 130 drugs and diseases from the 50 diseases. As the number of interacting drug-disease pairs is significantly fewer than noninteracting pairs in practical situations, the negative set was twice as large as the positive set. Then we performed a 10-fold cross-validation procedure in matrix factorization: the training samples were randomly split into 10 subsets of roughly equal size; each subset was in turn taken as the test set. The performance was evaluated by plotting a receiver operating characteristic (ROC) curve for the test set and calculating the Area under the ROC Curve (AUC). We repeated the cross-validation experiment independently for 5 times, in each of which a different random partition of the samples to 10 subsets was used, and took the average to obtain a robust AUC score estimate.

**Figure 2:**
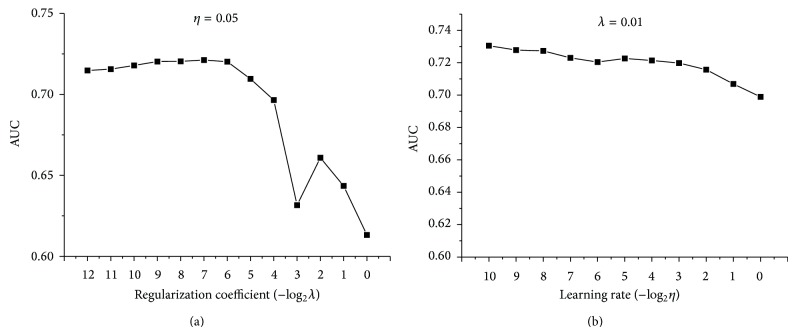
The AUC results of models based on different penalization and learning rate. (a) Learning rate remains the same, while penalization coefficients increase twofold each time. (b) Penalization coefficient remains the same, while learning rate increases twofold each time.

In the training, two coefficients have to be determined firstly: regularization coefficient and learning rate. Models based on different coefficients were set up and measured according to the performance of AUC. As illustrated in Figure 2, regularization coefficient exerts a large influence on the model, while learning rate has a relatively marginal effect. Smaller regularization coefficient means a better fitting of the training samples, while larger regularization would penalize more on overfitting. An appropriate regularization coefficient is chosen to reach a compromise between training error and generalization ability. Learning rate has a limited influence on the model. The performance decreases insignificantly with the increase of learning rate. Thus, a larger learning rate is preferred as it indicates a shorter training time. Finally, regularization coefficient was set as 2^−7^, and learning rate was set as 2^−4^.

**Figure 3:**
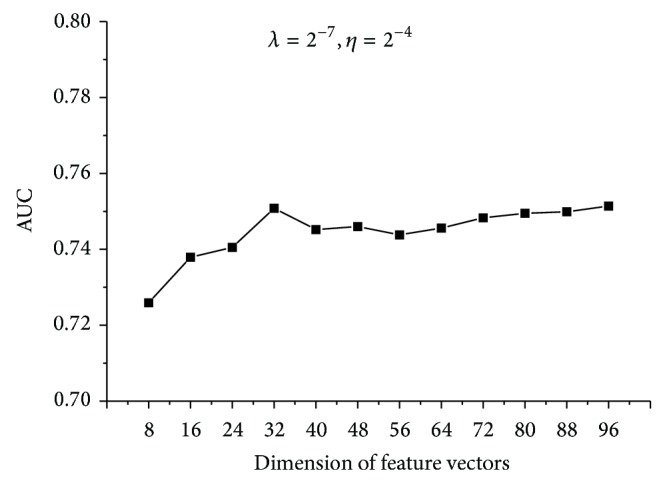
The AUC results of models based on different dimensions. Penalization coefficient and learning rate remain the same.

**Figure 4:**
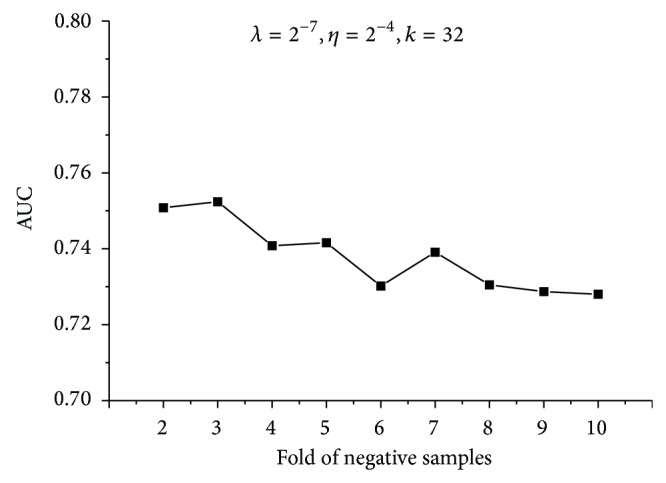
The AUC results of models based on different folds of negative samples. Penalization coefficient and learning rate remain the same. The dimension of feature vectors is set as 32.

It should be noted that the dimension of feature vectors, denoted by *k*, was set as 8 in the previous experiment. However, it is not a requirement. As the feature vectors were extracted from eigenvalue decomposition of the 994 × 994 gene relation matrix, *k* can be set as any value from 1 to 994. As illustrated in Figure 3, we changed the dimension of feature vectors and compared the performances of models. It can be seen that the chosen of *k* does not affect the performance much. Larger value of *k* should lead to less variance from the original data; nevertheless, the increase of AUC is quite small compared to the expansion of feature vectors. Thus, we chose *k* as 32, which results in an acceptable performance. Finally, we note that taking the negative set several times as large as the positive set has a negligible effect on the resulting AUC score, which is demonstrated in Figure 4.

To conclude, we set regularization coefficient as 2^−7^, learning rate as 2^−4^, and dimension of feature vectors as 32 and set the negative set twice as large as the positive set to generate our model.

### 3.3. Performance Results

It can be seen that our method is constituted by two main parts: the first part is to construct the feature vectors of genes, drugs, and diseases; the second part is to generate the matrix factorization model. In our method, the topological characteristics of gene interaction network are fused into matrix factorization by extracting feature vectors of genes, drugs, and diseases. To demonstrate the rationale for our method, we firstly compared the performance of our method with the two single parts. As to the first part, we denote it by “FV.” To get rid of matrix factorization, the dot products of feature vectors of drugs and diseases are utilized directly to measure their possibility of association. The detailed procedure is as follows.

1. Initiate states of the decomposed matrices *A* and *B* with extracted feature vectors from genomic space.
2. Calculate dot products of feature vectors of drugs and diseases to measure their possibility of association.
3. Perform 5 × 10-fold cross-validation and calculate the average AUC as a result.

As to the second part, we denote it by “MF.” The feature vectors of drugs and diseases are randomly initialized to eliminate topological characteristics of gene interaction network. The detailed procedure is as follows.

1. Randomly initiate states of the decomposed matrices *A* and *B*.
2. Train the matrix factorization model and use it to predict possibility of association between drugs and diseases.
3. Perform 5 × 10-fold cross validation and calculate the average AUC as a result.

The experiments were undertaken on the same cross-validation samples. The results are listed in Table 1. From the results, we can find that the performance of FV is lowest, which suggests that measurement of drug-disease associations which is solely based on gene interaction network is quite savage and impracticable. However, after topological characteristics of gene interaction network are fused into matrix factorization, the AUC result increases from 0.5778 to 0.7508. This exhibits the usefulness of genomic space in improving the performance of matrix factorization and verifies that drug-disease associations have implications in the gene interaction network.

Next, three widely used collaborative filtering tools, libFM [18], SVDFeature [19], and libMF [20] were also applied here, as competing methods, to predict drug-disease associations.

libFM transforms the drug-disease association into the form of *n*-dimensional feature vector *X* with its corresponding target *y*. Then a factorization machine is generated as follows:

(7) $$\begin{array}{c} \hat{y}\left(\;X\;\right)≔\omega_{0}+\overset{n}{\underset{i=1}{\sum }}\omega_{i}x_{i}+\overset{n}{\underset{i=1}{\sum }}\overset{n}{\underset{j=i+1}{\sum }}\langle \;v_{i},v_{j}\;\rangle x_{i}x_{j}, \end{array}$$

where the model parameters that have to be estimated are *ω*_0_ ∈ *ℝ*, *ω* ∈ *ℝ* ^*n*^, and **V** ∈ *ℝ* ^*n*×*k*^. A row **v**_*i*_ within **V** describes the *i*th variable with *k* factors. Finally, the output of the factorization machine can be used to predict possibility of association between novel drug-disease pairs.

SVDFeature extracts bias information and latent variables from drug-disease associations. For drugs, bias item *b*_*r*_ and latent feature vector *p*_*r*_ are estimated. For diseases, bias item *b*_*i*_ and latent feature vector *q*_*i*_ are estimated. Global bias is estimated as *b*_*g*_. Global mean value is denoted by *μ*. Other input includes *α* for drug feature, *β* for disease feature, and *γ* for global feature. Finally, the association between drug *r* and disease *i* is calculated as follows:

(8) $$\begin{array}{c} y_{ri}≔\mu +\left(\;b_{g}\gamma +b_{r}\alpha +b_{i}\beta \;\right)+{\left(\;p_{r}\alpha \;\right)}^{T}\left(\;q_{i}\beta \;\right). \end{array}$$

libMF estimates drug bias (*a*_*u*_), disease bias (*b*_*v*_), average term (avg), and latent factors (*p*_*u*_, *q*_*v*_) from the training samples and calculates the novel drug-disease pair as follows:

(9) $$\begin{array}{c} y≔\text{avg}+a_{u}+b_{v}+{p_{u}}^{T}q_{v}. \end{array}$$

We evaluated the predictive performance of the four methods in three settings: pair prediction, drug prediction, and disease prediction. In all settings, the negative set was twice as large as the positive set. In pair prediction, 5 × 10-fold cross-validation was performed and the test set was chosen randomly. The average AUC was taken as a result. To test the applicability of our method in predicting indications of new drug candidate compounds associated with no diseases or predicting drugs of orphan diseases, we performed drug prediction and disease prediction. In drug prediction, all the drugs were randomly partitioned into ten subsets of equal sizes. The associations regarding all the drugs in each subset were in turn chosen as the test set. Thus, all the drugs in the test set are outside of the training set. The procedure was repeated 5 times to calculate the average AUC result. Similarly, in disease prediction, all the diseases were randomly partitioned into ten subsets of equal sizes. The associations regarding all the diseases in each subset were in turn chosen as the test set. Also, the procedure was repeated 5 times to obtain the average AUC result. In the experiment, parameters in our method were set as follows: regularization coefficient is set as 2^−7^, learning rate is set as 2^−4^, and dimension of feature vectors is set as 32. Parameters in libFM were set as those in [18]. Parameters in SVDFeature were set as guided in [21]. Parameters in libMF were set as those in [20].

As illustrated in Table 2, our method outperforms the other three competing methods in all of the three settings. The introduction of genomic space improves the AUC performance, which implicates that the extraction of feature vectors might be quite favorable for the characterization of the topology of gene interaction network. From the comparison, it can be found that our method is reliable in predicting drug-disease associations. Particularly, our method performs better than the competing methods in drug prediction and disease prediction. The relatively bad performance in disease prediction can be attributed to the small quantity of disease-gene interactions. On average, each investigated disease has no more than two interacting genes. However, the performance of our method in disease prediction is still much better than the competing methods, which shows that the topology information from genomic space is effective in relating drugs to diseases in cases where association information of drugs or diseases is rare.

### 3.4. Evaluation on a Larger Dataset

**Figure 5:**
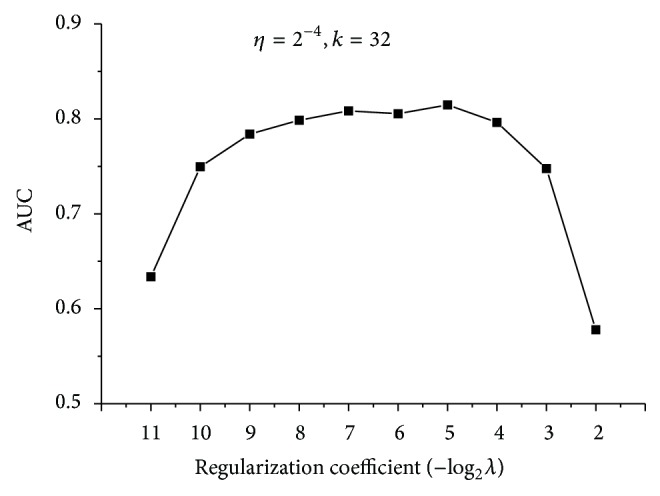
The AUC results of models based on different penalization coefficients. Learning rate remains the same. The dimension of feature vectors is set as 32.

To test the practicability of our method, we relaxed the requirement for preparation of interaction data and performed the experiment once again. On this occasion, a drug-disease association, in which either the drug or the disease has interactors in the 9415 genes, was also kept. In this way, 1076 drug-disease associations involving 398 drugs and 242 diseases were left, among which only 200 drugs and 70 diseases have interacting genes. Besides, 1222 drug-gene interactions concerning the 200 drugs and 97 disease-gene interactions concerning the 70 diseases were left. In total, there were 1059 distinct genes in these interactions. Based on these data, experiments of 5 × 10-fold cross-validations were undertaken for pair prediction, drug prediction, and disease prediction. In all settings, the negative set was twice as large as the positive set. In our method, as the influence of both learning rate and dimension of feature vectors on the performance is limited, the two parameters were set in the same way as that in the previous experiment. As shown in Figure 5, regularization coefficient exerts a larger influence on the performance. Here, the regularization coefficient was chosen as 2^−5^. Before training, the feature vectors of drugs and diseases that have no interacting genes were initialized randomly. Specifically, 198 drugs out of 398 and 172 diseases out of 242 were assigned with random feature vectors of the designated rank. Parameters of the three competing methods were set in the same way as that in the previous experiment.

Finally, the results are listed in Table 3. It can be seen that our method still performs better than the competing methods. However, the advantage is not that distinct as in the previous experiment. This is due to the small proportion of drugs (200/398) and diseases (70/242) that have interacting genes. Considering that large amounts of drugs and diseases which have no evidence from the genomic space, the performance of our method is quite remarkable. It can be expected that our method would perform much better when more and more interaction data are accumulated in the future.

## 4. Discussion

In this work, we proposed a matrix factorization model with the integration of genomic space to detect potential drug-disease associations and predict novel drug indications. Feature vectors of genes, drugs, and diseases were firstly retrieved from genomic space. In this way, the topological characteristics of gene interaction network were fused into the matrix factorization model.

We collected a set of pairwise binary interactions between genes to construct the gene interaction network. Based on the distances in the network, we set up the gene relation matrix, which was directly used to extract feature vectors of genes. Afterwards, feature vectors of drugs and diseases were obtained according to the interacting genes of them. Next, we adopted the matrix factorization model, which generates two low-rank matrices to reconstruct the known drug-disease associations. To constrain the output to fall within the range of 0-1, we added a sigmoid active function to the output. Stochastic gradient descent was used to learn the model, which was then employed in the prediction of novel drug-disease associations.

From the performance of FV and MF in Table 1, we can see that the incorporation of topological information of genomic space by initiating states of the decomposed matrices *A* and *B* with extracted feature vectors is effective. In a mathematical sense, this might be attributed to sensitivity of the model to the starting points during the training; and this might be the reason why the proposed model improves the predictive ability. Further investigation will be undertaken in our later work.

To date, most of the research relies on matching drug and disease profiles to detect potential drug-disease associations. Nevertheless, it takes time to prepare so many profiles for large amount of drugs and diseases. On the contrary, our method takes advantage of interaction data and is free of calculating profiles of drugs and diseases. It should be noted that our method is more tractable and applicable as the interaction data can be easily obtained from public database and directly employed to predict novel drug indications. Furthermore, our method relieves us of the bother of selecting appropriate combinations of features for drugs and diseases.

One major limitation of our method is that it relies heavily on the richness of interaction data. The availability of drug-gene interactions and disease-gene interactions is a key point for accurate measurement of feature vectors of drugs and diseases. In our research, the performance of our method has been affected by the small scale of interaction data, especially disease-gene interactions. However, it is not an unattractive option for the reason that it gets rid of the bother of selecting and collecting profiles for drugs and diseases; and the evaluation on the larger dataset has proven its validity in relatively large scale data, as the performance is acceptable even if large amounts of drugs and diseases have no interacting genes on that occasion. With the accumulation of more and more interaction data in the future, we believe that our method would play a much greater role in the prediction of novel drug indications.

## Figures and Tables

**Algorithm 1:**
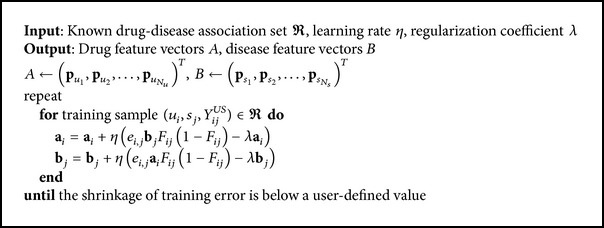
Algorithm for learning the matrix factorization model.

**Table 1:** Performance of FV and MF.

	Our method	FV	MF

AUC	0.7508	0.5264	0.5778

**Table 2:** AUC values obtained by 5 × 10-fold cross-validation.

	Pair prediction	Drug prediction	Disease prediction
libFM	0.7068	0.6637	0.349
SVDFeature	0.5699	0.6403	0.236
libMF	0.5868	0.6557	0.3173
Our method	0.7508	0.7216	0.532

**Table 3:** AUC values obtained by 5 × 10-fold cross-validation.

	Pair prediction	Drug prediction	Disease prediction
libFM	0.8078	0.6226	0.3682
SVDFeature	0.7107	0.6069	0.3338
libMF	0.6388	0.6217	0.3878
Our method	0.8147	0.6573	0.5617
